# I drink for my liver, Doc: emerging evidence that coffee prevents cirrhosis

**DOI:** 10.12688/f1000research.6368.2

**Published:** 2015-11-11

**Authors:** Jordan J. Feld, Élise G. Lavoie, Michel Fausther, Jonathan A. Dranoff

**Affiliations:** 1 Toronto Western Hospital Liver Center, Toronto, ON, M5G 2M9, Canada; 2 Division of Gastroenterology and Hepatology, University of Arkansas for Medical Sciences, Little Rock, AR, USA

**Keywords:** adenosine receptor, coffee, liver fibrosis, cirrhosis

## Abstract

Evidence demonstrating that regular ingestion of coffee has salutary effects on patients with chronic liver disease is accumulating rapidly. Specifically, it appears that coffee ingestion can slow the progression of liver fibrosis, preventing cirrhosis and hepatocellular carcinoma (HCC). This should excite clinicians and scientists alike, since these observations, if true, would create effective, testable hypotheses that should lead to improved understanding on fibrosis pathogenesis and thus may generate novel pharmacologic treatments of patients with chronic liver disease.

This review is designed to examine the relevant clinical and epidemiological data in critical fashion and to examine the putative pharmacological effects of coffee relevant to the pathogenesis of liver fibrosis and cirrhosis. We hope that this will inspire relevant critical analyses, especially among “coffee skeptics”. Of note, one major assumption made by this review is that the bulk of the effects of coffee consumption are mediated by caffeine, rather than by other chemical constituents of coffee. Our rationales for this assumption are threefold: first, caffeine’s effects on adenosinergic signaling provide testable hypotheses; second, although there are  myriad chemical constituents of coffee, they are present in very low concentrations, and perhaps more importantly, vary greatly between coffee products and production methods (it is important to note that we do not dismiss the “botanical” hypothesis here; rather, we do not emphasize it at present due to the limitations of the studies examined); lastly, some (but not all) observational studies have examined both coffee and non-coffee caffeine consumption and found consistent effects, and when examined, no benefit to decaffeinated coffee has been observed. Further, in the interval since we examined this phenomenon last, further evidence has accumulated supporting caffeine as the effector molecule for coffee’s salutary effects.

## Analysis of clinical and epidemiological data

### General comments

It was recognized decades ago that caffeine is a vasoactive molecule; this led to concerns that coffee consumption may be associated with an increased risk of cardiovascular diseases, potentially leading to an increased risk of all-cause mortality. An early study by Klatsky and colleagues (1993) to address this issue using the Kaiser-Permanente database found that although very high coffee intake (> 4 cups per day) was associated with a slightly increased risk of myocardial infarction (relative risk 1.4, 95% CI 1.0–1.9), there was no overall effect on mortality, largely due to an unexpected finding of fewer deaths due to cirrhosis in coffee drinkers than non-drinkers. With each additional cup of coffee consumed per day, the risk of death from cirrhosis fell by 23% (RR 0.77, 95% CI 0.67–0.89) ^4^. Subsequent studies have confirmed that coffee consumption is associated with improved outcomes on many parameters of liver disease ranging from liver enzyme levels and histological activity to rates of liver fibrosis progression and incidence of cirrhosis and hepatocellular carcinoma (HCC) ^1– 3^. The almost exclusively observational nature of the data has made it difficult to draw strong conclusions about causation and to identify the specific mechanisms involved; however, the consistency and magnitude of the findings certainly justify further investigations to clarify how coffee improves liver health.

### Coffee and liver enzyme elevations

Early studies from Europe and Japan found that regular coffee consumption was associated with lower gamma glutamyl transferase (GGT) and alanine aminotransferase (ALT) levels ^5– 9^. Ruhl and Everhart (2005) used data from the National Health and Nutrition Examination Survey (NHANES) to evaluate the association between coffee and caffeine intake and ALT elevation in American patients at increased risk for liver disease from alcohol, viral hepatitis, or obesity. They found a lower prevalence of ALT elevation with increasing coffee and particularly with increasing caffeine intake. After adjustment for confounders, individuals in the highest quintile of caffeine consumption had less than one third the risk of ALT elevation of those in the lowest quintile (odds ratio (OR) 0.31, 95% CI 0.16–0.61) ^10^. To explore possible explanations for their findings, they evaluated whether lower insulin resistance in coffee drinkers could account for the reduced ALT levels. Although coffee consumption was inversely associated with fasting insulin levels, the relationship between coffee or caffeine intake and ALT was unaffected by inclusion of insulin levels in the model ^10^. Thus, the possible effect of coffee on insulin/sugar balance was not a sufficient mechanism to explain the effects observed.

More recently, Molloy and colleagues (2012) evaluated the effect of coffee and caffeine consumption in patients with non-alcoholic fatty liver disease (NAFLD). They found a weak but statistically significant inverse correlation between caffeine consumption and ALT levels. Notably, caffeine and coffee intake were similar between patients without any evidence of NAFLD and those with established non-alcoholic steatohepatitis (NASH), whereas intake was lower in patients with NASH than in those with simple steatosis, suggesting that the protective effect of coffee and/or caffeine may be greatest in patients at risk for progressive liver disease ^11^.

Interestingly, in patients with chronic hepatitis C virus (HCV) infection, no relationship between coffee or caffeine consumption and ALT levels has been observed, despite the fact that increasing intake was found to be associated with reduced histological activity and fibrosis on liver biopsy ^11, 12^. This observation raises the possibility that coffee and/or caffeine consumption directly inhibit hepatic fibrosis independent of reducing hepatic inflammation, providing a distinct rationale for the study of coffee/caffeine on liver fibrogenic mechanisms.

### Coffee and liver fibrosis in humans

More important than an effect on aminotransferase levels, increasing coffee and caffeine consumption has been found to be associated with reduced liver fibrosis, a finding that has been largely consistent across studies in HCV and fatty liver disease, whether related to NASH or alcohol.

Initial studies from Italy found that patients with cirrhosis consumed less caffeine, and specifically less caffeine from coffee, than age and sex-matched controls ^13^. Odds ratios for presence of cirrhosis in this study increased as a function of coffee consumption: 0.47 (95% CI 0.20–1.10) for patients consuming 1 cup of coffee per day and 0.16 (95% CI 0.05–0.50) for patients consuming 4 cups per day. Here the reference against which the above groups are compared is lifetime coffee abstainers. Caffeine intake from sources other than coffee was similar between cases and controls; however, it is critical to note that coffee accounted for the vast majority of caffeine consumption in both groups (likely reflecting the dietary habits of the Italian population studied). Similar results were seen in other studies using a case-control design ^14, 15^.

Modi and colleagues evaluated a cohort of patients with chronic liver diseases of various etiologies and found that patients with advanced fibrosis consumed less coffee and less caffeine than those with milder liver damage ^12^. The effect size was greatest in patients with chronic HCV infection. They also found no relationship between caffeine from sources other than coffee or intake of decaffeinated coffee and the severity of liver fibrosis. Coffee, and caffeine specifically, is metabolized almost exclusively within the liver, which has raised the issue that individuals with more advanced liver fibrosis may reduce coffee intake because of a greater clinical effect of lower doses with progressive hepatic impairment. It is also possible that individuals with more advanced liver disease reduce coffee intake due to a perception that coffee is unhealthy. Modi and colleagues (2010) found that results from caffeine consumption questionnaires were consistent over time, and patients with more advanced fibrosis did not report reducing coffee or caffeine consumption as their disease progressed ^12^.

To assess the clinical significance of fibrosis progression, Freedman and colleagues (2009) evaluated the effect of coffee consumption in the large HALT-C study, which included only patients with bridging fibrosis (F3) or cirrhosis (F4) ^16^. They found that at baseline, increased coffee consumption was associated with milder liver disease; perhaps more importantly, during the 4-year study period, they found that patients who consumed more coffee had a lower risk of experiencing adverse clinical outcomes. Patients who consumed no coffee had a risk of hepatic decompensation or HCC of 11.1 per 100 patient-years compared to just 6.3 per 100 patient-years in those consuming ≥ 3 cups per day. Once again, no beneficial effect was seen with tea or other sources of caffeine. Interestingly, coffee consumption was also associated with better clinical responses to peginterferon and ribavirin therapy in this cohort ^17^.

Coffee has been shown to be associated with less severe fibrosis in patients with NASH as well. Interestingly, although coffee consumption was associated with less severe hepatic steatosis, the effect may not be limited to liver injury ^11^. Increasing coffee consumption was found to be associated with a lower risk of metabolic syndrome in Japanese men, particularly in those drinking ≥ 4 cups per day (OR 0.61, 95% CI 0.39–0.95). The reduced rate of metabolic syndrome was due to an inverse association between coffee consumption and both blood pressure and triglyceride levels after controlling for other relevant factors ^18^. Large population-based studies have also found that increasing coffee intake is associated with a lower incidence of diabetes ^19– 21^. The recent finding that coffee consumption was associated with a lower risk of insulin resistance and liver fibrosis in patients with HIV-HCV co-infection raises the possibility that the beneficial hepatic effects of coffee on the liver may relate to improved metabolic parameters, even in patients with diseases other than NAFLD ^22^.

Overall, observational data have consistently shown that patients with more advanced liver fibrosis consume less coffee than those with milder disease, particularly in patients with HCV and NAFLD. Although these data are certainly suggestive of a clinical benefit of coffee on fibrosis progression, caution must be taken before drawing direct causal inferences from these observational, non-interventional studies.

### Coffee and HCC

The initial observation that increased coffee consumption was associated with a lower incidence of HCC came from epidemiological studies from Italy and Greece ^14^. This finding has been confirmed in multiple subsequent studies, including meta-analyses from other parts of the world ^23, 24^. Reassuringly, similar effects have been seen in case-control and cohort studies. The most recent meta-analysis including 16 studies with 3153 cases of HCC found that coffee consumption was associated with an overall relative risk of 0.60 (95% CI 0.50 to 0.71) for HCC compared to those who drink no coffee at all ^25^. The results were consistent across studies after controlling for confounders and importantly showed that the apparent benefits of coffee seemed to increase with each additional cup consumed per day (RR of 0.80 per cup per day). Cirrhosis is the single most important risk factor for HCC. Whether coffee directly affects hepatic carcinogenesis or reduces HCC by slowing the progression of fibrosis and development of cirrhosis remains unclear.

### What is the active anti-fibrotic ingredient in coffee?

There are as many as 1000 substances in coffee, any of which may have hepatoprotective or anti-fibrotic properties. Most studies have focused on caffeine, diterphenoic alcohols (cafestol and kawheol), as well as possible antioxidant properties of chlorogenic acid and tocopherols. To date, no studies have found an association between caffeine consumption from sources other than coffee and reduced liver injury. However, in almost all epidemiological studies to date, the vast majority of caffeine in the diet came from coffee consumption. To achieve equivalent levels of total caffeine intake, individuals must consume much more tea or caffeinated soda than coffee. Particularly if, as suggested in some studies ^12^, there is a threshold of caffeine intake for a beneficial effect, it may be difficult to reach this level from non-coffee sources of caffeine (see Table 2).

**Table 1.** Summary of scientific papers examining effects of coffee on human liver injury, fibrosis, and HCC.

Author/Year	Disease	Beverage	Coffee/Caffeine dose	Clinical effect
Liver Enzymes				
Casiglia/1993	None	Coffee	3 cups per day	Lower mean ALT/GGT/bilirubin among coffee drinkers
Honjo/2001	None	Coffee	1 to >5 cups/d	Stepwise decrease in risk of elevated ALT with each cup of coffee per day
Poikolanien/1997	None	Boiled or Filtered coffee	4–6 cups/d >7 cups/d	Reduced likelihood of elevated GGT. Greater effect with filtered coffee.
Tanaka/1997	None/Alcohol	Coffee	0 to >5 cups per day	Lower mean GGT/ALT with each cup of coffee. No effect of green tea.
Ruhl/2005	Alcohol/NAFLD/Viral hepatitis	Coffee/Total caffeine	0 to 20 cups per day	Lower ALT with increasing coffee or caffeine intake.
Modi/2010	HCV	Coffee/Total caffeine	0 to 1022 mg caffeine per day	No correlation between ALT and coffee or caffeine consumption
Liver Fibrosis				
Molloy/2012	NAFLD	Coffee/Total caffeine	0 to 822 mg caffeine per day	Increased coffee associated with reduced risk of NASH and fibrosis. No effect seen with other sources of caffeine.
Modi/2010	HCV	Coffee/Total caffeine	0 to 1022 mg caffeine per day	Reduced fibrosis seen in patients with higher coffee consumption.
Corrao/1994	Cirrhosis	Coffee	0 to >4 cups per day	Reduced odds of cirrhosis with increasing coffee intake
Freedman/2009	HCV (F3/F4)	Coffee	0 to >5 cups per day	Reduced hepatic decompensation with increased coffee intake
Hepatocellular carcinoma				
Gallus/2002	HCC	Coffee	0 to >3 cups per day	Reduced odds of developing HCC among coffee drinkers
Larsson/2007	HCC	Coffee	Meta-analysis	Consistent reduced risk of HCC among coffee drinkers. Limited or no effect with other sources of caffeine.
Bravi/2013	HCC	Coffee	Meta-analysis	Relative risk of 0.6 for HCC among coffee drinkers vs. non-drinkers

**Table 2.** Estimated caffeine doses of commonly ingested caffeinated substances ^59– 61^.

Beverage	Size	Caffine dose
**Coffee**	16 oz	140–240 mg
**Espresso**	1 shot	58–75 mg
**Decaf Coffee**	16 oz	<10 mg
**Black Tea**	12 oz	70–75 mg
**Green Tea**	8 oz	27–36 mg
**Cola Beverages**	12 oz	34–72 mg
**Caffeine caplets**	1 caplet	200 mg

Coffee preparation affects the composition of the final product. Interestingly, the apparent benefits of coffee may be greatest with filtered coffee. Drip coffee reduces cafestol and kawheol, which have been associated with increasing LDL cholesterol and possibly with increased ALT levels ^26^. This difference was borne out in a recent study that found that increasing filtered coffee consumption but not espresso consumption was associated with lesser degrees of liver fibrosis in obese European patients. In this study, espresso intake was associated with lower HDL cholesterol levels, higher triglyceride levels and a higher prevalence of metabolic syndrome ^27^. In response to a recent report documenting an association of coffee consumption with reduced total and cause-specific mortality ^28^, Aubin and Berlin noted that the benefits were largely seen in the era of filtered coffee consumption and may not extend to espresso and other unfiltered coffee, products which are increasing in use globally ^29^. This is further compounded by the high degree of variability between coffee preparations, with up to 6-fold differences in caffeine content between different commercially available espresso products ^30^. Clearly, before interventional studies can be seriously considered, it will be critical to clarify what in coffee has a hepatoprotective effect *and* what dose would be safe and effective.

### Summarizing the body of epidemiological data

Collectively the epidemiological data showing a beneficial association between increasing coffee consumption and severity of liver disease are strong. The consistency of the findings across different parameters of liver injury and in different liver diseases is reassuring. Importantly, coffee consumption has been associated not only with reduced liver fibrosis but also with a lower incidence of liver cancer and hepatic decompensation, which are critically relevant clinical outcomes. However, it is important to recognize some important limitations to the existing literature.

Specifically, the data are almost exclusively observational, and most studies have been cross-sectional in nature. Presumably a beneficial effect of coffee on liver disease would require prolonged exposure from early in the disease state to prevent progression, unless coffee somehow promotes fibrosis regression. As a result, studies finding an association between current coffee consumption and the current degree of liver fibrosis are limited due to a lack of accurate data on prior coffee intake. Although some studies have assessed the consistency of coffee intake over time, recall bias is still a major potential confounder. In addition, the possibility that patients with more advanced liver disease reduce their coffee intake over time specifically because of the severity of their liver disease must be considered, at least in part because they are often encouraged to reduce alcohol and tobacco use, both of which are highly correlated with coffee intake.

Unfortunately, it is difficult, if not impossible, to perform controlled trials of coffee use with hard clinical endpoints, most of which take years to occur. Cardin and colleagues (2013) recently performed a crossover-controlled trial of filtered coffee intake (4 cups per day) compared to none over a 30-day period in patients with chronic HCV infection. They found that during the period of coffee drinking, AST levels decreased, but GGT and HCV RNA levels rose. They also found that 8-dyrdoxydeoxyguanosine (8-OHDG) levels decreased, and telomere length increased, which they interpreted to suggest less oxidative DNA damage ^31^. Although the authors should be commended for trying to perform a controlled trial of coffee in patients with any chronic liver disease, it is hard to interpret the results. Numerous comparisons were made, and even those that were statistically significant were of questionable clinical importance. In addition, the biological plausibility is somewhat questionable given the short duration of the study. Overall, this study highlights the challenge of conducting controlled trials of dietary interventions.

### Coffee in animal models of liver fibrosis

As suggested above, there are exciting data from patients to suggest that coffee and/or caffeine prevent liver fibrosis; however, the cellular mechanisms by which this effect may work are not fully understood. In an attempt to elucidate these potential mechanisms, we will first examine some of the animal studies in which coffee and caffeine have been used in experimental models.

Whether caffeine or filtered coffee itself has been studied in rodent liver fibrosis/cirrhosis models (dimethylnitrosamine (DMN), carbon tetrachloride (CCl _4_) or thioacetamide (TAA)), fibrosis has been attenuated ^32– 37^. Interestingly, one trial examining Turkish-style coffee, which is unfiltered, demonstrated that liver fibrosis was not decreased and aminotransferase levels were *increased* in animals receiving CCl _4_ and Turkish coffee ^38^. It is important to note, however, that detailed mechanistic studies for the potentially beneficial effects of coffee in animal models of liver fibrosis have not been performed.

### Protection from fibrosis in animal models: coffee or caffeine?

One way that researchers have attempted to distinguish effects of coffee *vs* those of caffeine is through the use of trials in which decaffeinated coffee and/or non-coffee caffeine have been administered ^32, 35, 38^. The effect of non-coffee caffeine was protective against experimental liver fibrosis in three trials ^35, 38, 39^. However, two trials showed that decaffeinated coffee was also antifibrotic, albeit to a lower extent than caffeinated coffee ^32, 38^. We have interpreted these trials as part of a work in progress. Although the main effect of coffee as an antifibrotic in animals receiving experimental pro-fibrotic agents is largely mediated by caffeine, it is necessary for more, well-designed experiments to be performed.

### Caffeine as an antagonist of adenosine receptors

Caffeine has varied pharmacological effects, but one of its potent and best characterized effects is inhibition of adenosine receptors (AR) ^40^. There are four G protein-coupled receptors for extracellular adenosine: A _1_AR, A _2a_AR, A _2b_AR, and A _3_AR, each of which has its own signal transduction mechanism and downstream physiologic effects ^41, 42^. In addition, affinity for each receptor for adenosine varies as well: the high affinity receptors A _1_AR, A _2a_AR and A _3_AR are activated by low concentrations (>10 nM) of extracellular adenosine, whereas the low affinity A _2b_AR requires adenosine concentrations likely activated only in the setting of cell injury or death (>1 µM) ^43^.

In the liver, one of the most studied functions of adenosine is its protective role against ischemia/reperfusion, with potential implication of A _1_AR ^44^ and A _2a_AR ^45, 46^. The receptor that seems to be mainly responsible for adenosine protection is A _2b_AR ^47, 48^. A _1_AR was also shown to have a protective effect against ethanol-induced hepatotoxicity ^49^ and to protect against alpha-naphthylisothiocyanate-induced cholestatic liver injury induced by DPCPX (a specific A _1_AR antagonist) in A _1_AR deficient mice ^50^. A _2a_AR is expressed by heptatic stellate cells, where it regulates fibrogenesis and contractility ^51, 52^. A _1_AR and A _2a_AR antagonists were also shown to inhibit the protective effect of caffeine on portal hypertension-related complications ^53^. A _3_AR is overexpressed in hepatocellular carcinoma cells, and its activation is linked to apoptosis ^54^. A _3_AR agonists were shown to have anti-cancer properties *in vitro* and *in vivo* in the rat ^55^. These agents are currently studied in ongoing clinical trials ^56^. The same agents were also shown to have a protective effect against liver inflammation due to concanavalin-A injection in rats ^55^. Thus some adenosine receptor antagonists, like coffee/caffeine, may act against liver inflammation and fibrosis.

### Summary of studies examining coffee and liver injury/fibrosis in animal models

The data presented in this section support the concept that, in well-established models of liver fibrosis in animals (almost exclusively rodents), coffee provides a protective effect. Until a better hypothesis is tested, we may conclude that the protective effect occurs at the level of HSC A _2a_AR, with caffeine acting as an inhibitor. An alternative possibility is worth considering, however. Specifically, caffeine may be blocking inflammation rather than fibrosis directly, since adenosinergic signaling in inflammatory cells is well-established ^57, 58^. In addition, it is naïve to assume that rodent models of liver fibrosis/cirrhosis, such as CCl _4_, are effective analogues of human diseases, such as viral hepatitis and alcoholic liver disease. That said, these models are strong *in vivo* tests of liver myofibroblastic function, so they are essential steps in the testing of coffee and caffeine testing in cirrhosis pathogenesis.

## Conclusion

It seems very likely that coffee, acting through caffeine, and probably through inhibition of adenosinergic signals, prevents complications of chronic liver disease – specifically cirrhosis. Two features of the evidence are of particular importance. First, the fact that the literature in patients supporting coffee’s anti-cirrhotic effect continues to accrue without opposing studies suggests that the initial epidemiological associations were real. Although this could be accounted for in part by publication bias favoring positive studies, that is not a fully convincing explanation. Second, the observation that the studies in human are supported by animal and cellular data suggest that there is a rationale to give the human trials greater consideration. At present, it is rational to encourage the use of moderate amounts of brewed coffee in patients with chronic liver disease.
