# Supplementary material for: I drink for my liver, Doc: emerging evidence that coffee prevents cirrhosis
Source: F1000Res. 2015 Nov 11;4:95. Originally published 2015 Apr 21. [Version 2] doi: 10.12688/f1000research.6368.2 (PMC4416533; doi:10.12688/f1000research.6368.2)
Supplement: Peer review history

# Peer review: Referee response for version 1

**DOI:** 10.5256/f1000research.6828.r8510

**Published:** 2015-04-29

**Authors:** Vijay H. Shah

**Affiliations:** 1 Gastroenterology and Hepatology, Mayo Clinic, Rochester, MN, USA

This is a well-written review about beneficial effects of coffee on fibrogenesis and liver injury encompassing both lab and clinical studies. A couple points warrant the authors’ attention:

1. Caffeine also regulates phosphodiesterases. Could this be a contributory factor for beneficial effects?
2. Carbonated beverages also have significant levels of caffeine. Not aware of their benefits for fibrosis though. Any literature about this in context of fibrosis to support or refute the concept of caffeine as the antifibrotic component of coffee?

I have read this submission. I believe that I have an appropriate level of expertise to confirm that it is of an acceptable scientific standard.

# Response

**Published:** 2015-04-29

**Authors:** Jonathan A Dranoff

**Affiliations:** Division of Gastroenterology and Hepatology, Department of Internal Medicine, University of Arkansas for Medical Sciences, USA

We thank the reviewer for his kind comments.

1. Caffeine has several biochemical effects, including phosphodiesterase (PDE) inhibition. If the primary PDE inhibited were a regulator of cAMP (as is generally accepted), our collaborative work with Waj Mehal's group would suggest that caffeine might hasten liver fibrosis progression, since adenosine and its downstream effector cAMP are profibrogenic *in vitro*.
2. I am not aware of any beneficial effects of carbonated beverages; however, the relative caffeine content of carbonated beverages is quite low relative to drip coffee. Moreover, in the case of sugar-containing soft drinks, potential benefits of caffeine would be likely to be masked by the adverse metabolic effects of simple sugars. Note also that the relatively low caffeine content even of black and oolong teas (green and white teas have even less caffeine) probably accounts for the lack of epidemiological data supporting their beneficial effects in liver fibrosis progression, if caffeine is indeed the active ingredient.

# Peer review: Referee response for version 1

**DOI:** 10.5256/f1000research.6828.r8403

**Published:** 2015-04-27

**Authors:** Kinji Asahina

**Affiliations:** 1 Southern California Research Center for ALPD and Cirrhosis, Department of Pathology, Keck School of Medicine, University of Southern California, Los Angeles, CA, USA

This is an excellent review covering recent findings on beneficial effects of coffee on liver cirrhosis. The authors reviewed the relevant clinical and epidemiological data and suggested an inhibitory role of caffeine on adenosinergic signaling in hepatic stellate cells.

1.  Caffeine inhibits adenosine receptors. Contrary to this notion, the authors mentioned that antagonists for the receptors inhibit the protective effect of caffeine on portal hypertension-related complications on page 6. Please check whether antagonists for adenosine receptors inhibit the beneficial effects of caffeine in the liver.  
2. Are there papers showing adenosine receptor signaling in hepatic stellate cells? Does the inhibition the receptor suppress activation of hepatic stellate cells or induce their cell death?
3. Caffeine has been shown to induce autophagy in hepatocytes. Is it possible that activation of autophagy in hepatocytes indirectly mediates the activation state of hepatic stellate cells in the liver? 
4. Please indicate Table 1 in the text.

I have read this submission. I believe that I have an appropriate level of expertise to confirm that it is of an acceptable scientific standard.

# Response

**Published:** 2015-04-27

**Authors:** Jonathan A Dranoff

**Affiliations:** Division of Gastroenterology and Hepatology, Department of Internal Medicine, University of Arkansas for Medical Sciences, USA

Please see my answers to the questions above.

1. This is a reasonable question, but one that is currently unanswered.
2. There are indeed good data to demonstrate the presence of functional adenosine receptors in myofibroblastic HSC. Please see our collaborations with Waj Mehal's group.
3. This is certainly possible, but it has not been examined directly. Natalie Torok's work shows elegantly that HSC can endocytose apoptotic hepatocyte remnants, so this is worth examining.
4.  I am not sure that I understand - both tables are labeled and described.

Thanks for the supportive review.

# Peer review: Referee response for version 1

**DOI:** 10.5256/f1000research.6828.r8419

**Published:** 2015-04-22

**Authors:** Heather Francis

**Affiliations:** 1 Department of Medicine, Scott And White Digestive Research Center, Texas A&M Health Science Center College of Medicine, Temple, TX, USA

This is an excellent review regarding an area of some controversy, the beneficial effects of coffee/caffeine on liver disease. Feld, *et al*. have presented recent studies from both human populations as well as animal studies that provide more mechanistic data. The review is concise and highlights important work without bogging the reader down in too much detail. From this review, it's clear that more work needs to be done to fully understand the potential benefits of coffee and caffeine on liver fibrosis and other liver diseases.

I have read this submission. I believe that I have an appropriate level of expertise to confirm that it is of an acceptable scientific standard.

# Response

**Published:** 2015-04-27

**Authors:** Jonathan A Dranoff

**Affiliations:** Division of Gastroenterology and Hepatology, Department of Internal Medicine, University of Arkansas for Medical Sciences, USA

We greatly appreciate your kind review. We hope that two things arise from such a review:

1. Investigators are encouraged to engage in new research projects related to this work.
2. This will serve as a firm starting-point for those hoping to critically review this topic.
